# Safety and efficacy of direct oral anticoagulants in stroke prevention in patients with atrial fibrillation complicated with anemia and/or thrombocytopenia: a retrospective cohort study

**DOI:** 10.1186/s12959-023-00563-7

**Published:** 2023-11-21

**Authors:** Wenlin Xu, Jiana Chen, Shuyi Wu, Nianxu Huang, Xia Chen, Wang Zhang, Wei Hu, Jun Su, Hengfen Dai, Ping Gu, Xiaohong Huang, Xiaoming Du, Ruijuan Li, Qiaowei Zheng, Xiangsheng Lin, Yanxia Zhang, Lang Zou, Yuxin Liu, Min Zhang, Xiumei Liu, Zhu Zhu, Jinhua Zhang

**Affiliations:** 1 Department of Pharmacy, Fujian Maternity and Child Health Hospital College of Clinical Medicine for Obstetrics & Gynecology and Pediatrics , Fujian Medical University, #18 Daoshan Road, Fuzhou, 350001 China; 2 Department of Pharmacy, Taikang Tongji (Wuhan) Hospital, Wuhan, 430000 China; 3 Chendu Qingbaijiang Maternal & Child Health Care Hospital, Chengdu, China; 4 Department of Pharmacy, The First People’s Hospital of Changde City, Changde, Hunan 415000 China; 5 Department of Pharmacy, Xinyang Central Hospital, Xinyang Hospital Affiliated to Zhengzhou University, Xinyang, Henan 464000 China; 6 Department of Pharmacy, The First Affiliated Hospital of Bengbu Medical College, Bengbu, Anhui 233004 China; 7 Department of Pharmacy, Affiliated Fuzhou First Hospital of Fujian Medical University, Fuzhou, Fujian 350009 China; 8 Department of Pharmacy, Suining Central Hospital, Suining, Sichuan 629000 China; 9 Department of Pharmacy, Zhangzhou Affiliated Hospital of Fujian Medical University, Zhangzhou, Fujian 363000 China; 10 Department of Pharmacy, Shengjing Hospital of China Medical University, Shenyang, 110004 China; 11 Department of Pharmacy, Shanxi Bethune Hospital, Shanxi Academy of Medical Sciences, Tongji Shanxi Hospital, Third Hospital of Shanxi Medical University, Taiyuan, Shanxi 030032 China; 12 Department of Pharmacy, First Affiliated Hospital of Xi’an Jiaotong University, Xi’an, Shaanxi 710061 China; 13 Department of Pharmacy, Pingtan County General Laboratory Area Hospital, Fuzhou, Fujian 350400 China; 14 Department of Pharmacy, The First Affiliated Hospital of Jiamusi University, Jiamusi, Heilongjiang 154002 China; 15 Department of Pharmacy, Second Affiliated Hospital, Army Medical University, Chongqing, 400037 China; 16 Department of Pharmacy, Huaihe Hospital of Henan University, Kaifeng, Henan 475000 China; 17 Department of Pharmacy, Affiliated Qingdao Third People’s Hospital, Qingdao University, Qingdao, Shandong 266041 China; 18 Department of Pharmacy, People’s Hospital of He’nan University of Chinese Medicine (People’s Hospital of Zhengzhou), Zhengzhou, China; 19 Department of Pharmacy, The Second Affiliated Hospital of Soochow University, Suzhou, Jiangsu 215004 China

**Keywords:** Anemia, Atrial fibrillation, Hemoglobin, DOACs, Platelet, Thrombocytopenia

## Abstract

**Background:**

There are limited data about the clinical benefits and harm of direct oral anticoagulants (DOACs) in stroke prevention in patients with atrial fibrillation (AF) complicated with anemia or thrombocytopenia.

**Methods:**

This is a multi-center retrospective cohort study involving 5469 AF patients from 15 hospitals in China. Patients were divided into three groups according to hemoglobin and platelet levels: Group 1 (hemoglobin male ≥ 130 g/L; female ≥ 120 g/L and platelet ≥ 100 × 10^9^/L), Group 2 (hemoglobin male < 130 g/L; female < 120 g/L or platelet < 100 × 10^9^/L), and Group 3 (hemoglobin male < 130 g/L; female < 120 g/L and platelet < 100 × 10^9^/L). Patients in each category are further divided into two groups according to their stroke prevention strategies: rivaroxaban or dabigatran. Clinical results include major, minor, total bleeding, thrombosis, and the composite outcome of major bleeding and thrombosis.

**Results:**

Higher hemoglobin levels were associated with a reduced risk of total bleeding and major bleeding, while platelet counts were not associated with any event. Compared with Group 1, Group 2 had a higher risk of major bleeding (aOR 1.70, 95%CI 1.12–2.57, *P* = 0.012), and the composite endpoint of major bleeding and thrombosis (aOR 1.70, 95%CI 1.19–2.44, *P* = 0.004). Compared with Group 1, Group 3 had a higher total bleeding risk (aOR 2.15, 95%CI 1.14–4.05, *P* = 0.018). Compared with dabigatran, rivaroxaban was associated with higher composite risk in Group 1 (aOR 2.91, 95% CI 1.66–5.16, *P* < 0.001) and Group 2 (aOR 3.05, 95%CI 1.46–6.39, *P* = 0.003), but there was no significant difference in Group 3 (aOR 1.78, 95%CI 0.23—13.54, *P* = 0.577).

**Conclusions:**

Higher hemoglobin levels are associated with a reduced risk of total bleeding and major bleeding in patients with AF. Dabigatran was associated with better clinical outcomes than rivaroxaban in patients with anemia or thrombocytopenia but not in those with anemia and thrombocytopenia.

**Supplementary Information:**

The online version contains supplementary material available at 10.1186/s12959-023-00563-7.

## Introduction

Atrial fibrillation (AF) is a clinically common arrhythmia associated with increased morbidity and mortality as it increases the risk of stroke by 4 to 5-fold [1, 2]. Although CHA2DS2-VASc score (congestive heart failure, hypertension, age ≥ 75 years, diabetes mellitus, previous stroke or transient ischemic attack [TIA], vascular disease, age 65–74 years, female) [3] and HAS-BLED score (hypertension, abnormal renal or liver function, stroke, bleeding history, unstable INR, age ≥ 65 years and antiplatelet drugs or alcohol use) [4] are widely used for risk stratification of thrombosis or bleeding in patients with nonvalvular AF(NVAF). However, other clinical features not included in these scores may be risk factors for adverse outcomes such as thrombosis and bleeding in NVAF patients (such as low body weight or body mass index (BMI) [4, 5], low creatinine clearance (CrCl) [6, 7], etc.). Anemia is common in patients with AF, which may be a sign of occult bleeding. Indeed, anemia has been identified as a strong predictor of bleeding in AF patients treated with anticoagulants [8–11]. Anemia is also associated with increased rates of thromboembolic events in certain populations [12–14], including patients with AF [15]. In addition, previous studies in patients with AF have also shown that abnormal hemoglobin and platelet counts may be associated with adverse events. However, current studies examining the association between hemoglobin and platelet levels and adverse outcomes have reported conflicting data [16–19].

Oral anticoagulation therapy is recommended for AF patients with stroke risk factors to reduce the risk of stroke or systemic embolism [20]. Although the effect of direct oral anticoagulants (DOACs) is no less than or even better than that of warfarin [21, 22], several major randomized controlled trials of DOACs exclude patients with hemoglobin < 10 g/dL or platelet count < 90–100 K/μL [23–26]. Two previous studies [27, 28] showed that the bleeding risk of DOACs was lower than that of warfarin in AF patients with hemoglobin < 10 g/dL or platelet count < 100 × 10^3^/µL, while there was no difference in the risk of ischemic stroke/systemic embolism or death, but there was no comparison among DOACs. The available data on the clinical benefits and harms of DOACs in high-risk populations with AF and anemia or thrombocytopenia remain limited, and physicians face a therapeutic dilemma when selecting DOACs for AF patients with anemia or thrombocytopenia. Therefore, this study aimed to assess the association of clinical event risk with hemoglobin levels and platelet counts in patients with AF. In addition, we studied the clinical outcomes of different DOACs in different hemoglobin and platelet stratification.

## Methods

### Study design

From January 2016 to December 2020, we conducted retrospective multi-center registrations at 15 centers in China (Supplemental Table 1). The distribution of hospitals is shown in Supplementary Figure 1. The Ethics Committee of Fujian Provincial Maternity and Children’s Hospital approved the scheme (registration number: ChiCTR2300067734). Due to the retrospective nature of this study, the institutional review board waived the patient informed consent requirement. Patients included in this study must meet all of the following inclusion criteria: (1) Age ≥ 18 years; (2) Patients diagnosed with AF; (3) DOACs therapy. Exclusion criteria were as follows: (1) patients with valvular AF (VAF); (2) Baseline data lacked hemoglobin and platelet counts. Since Apixaban has no AF indication in China, and Edoxaban was only launched in China in 2019, DOACs used in multi-center hospitals only include Rivaroxaban and Dabigatran.

After meeting the inclusion criteria, 5469 NVAF patients treated with DOACs were eligible for this study. Patients were divided into three groups based on their hemoglobin levels and platelet counts: Group 1 (hemoglobin male ≥ 130 g/L; female ≥ 120 g/L and platelet ≥ 100 × 10^9^/L), Group 2 (hemoglobin male < 130 g/L; female < 120 g/L or platelet < 100 × 10^9^/L), and Group 3 (hemoglobin male < 130 g/L; female < 120 g/L and platelet < 100 × 10^9^/L). Anemia was defined according to the World Health Organization criteria as hemoglobin concentration < 130 g/L for men and < 120 g/L for women [29]. Each group was further graded according to the type of DOACs, namely rivaroxaban or dabigatran.

### Data collection

We collected demographic data through hospital information systems and obtained clinical events through follow-ups of patients or relatives. We collected basic data such as age, sex, height, and weight. At the same time, we collected information on comorbidities such as hypertension, diabetes, coronary heart disease, and heart failure and biochemical indicators such as alanine aminotransferase (ALT), creatinine, platelet count, and hemoglobin. Based on the patient’s clinical data, We performed CHA2DS2-VASc score [3] (congestive heart failure, hypertension, age ≥ 75 years, diabetes mellitus, previous stroke or TIA, vascular disease, age 65–74 years, female) and HAS-BLED score [4] (hypertension, abnormal renal or liver function, stroke, bleeding history, unstable INR, age ≥ 65 years and antiplatelet drugs or alcohol use).

### Study outcomes

The safety outcomes of this study were total bleeding, major bleeding, and minor bleeding. The effective outcome was a thrombotic event. Total bleeding includes all bleeding events, including major and minor bleeding. The International Society for Thrombosis and Hemostasis (ISTH) defines major bleeding as occurring in critical organs (intracranial, spinal, intraocular, retroperitoneal, intraarticular or pericardial, intramuscular compartment syndrome) or a decrease in hemoglobin levels of at least 2 g/dl or a transfusion of at least 2 units of red blood cells [30]. Minor bleeding events were defined as bleeding that did not meet the criteria for major or clinically significant bleeding. Thromboembolic events include ischemic stroke and systemic embolism. Systemic embolism is defined as acute vascular occlusion of a limb or organ documented by imaging, surgery, or autopsy [23].

### Statistical analysis

Continuous variables are expressed as mean and standard deviation (SD) or median and interquartile distance [IQR], while discrete variables are expressed as quantity and percentage. For comparison among the three groups, unpaired two-tailed T-tests or one-way analysis of variance (ANOVA) were used to assess differences between successive values. Differences between nominal variables were compared by Chi-square test. Logistic regression analysis was used to compare the risk of clinical events in different hemoglobin levels and platelet count groups and adjusted according to age; sex; CHA2DS2- VASc score (congestive heart failure, hypertension, age ≥ 75 years, diabetes mellitus, previous stroke or TIA, vascular disease, age 65–74 years, female); HAS-BLED score (hypertension, abnormal renal or liver function, stroke, bleeding history, unstable INR, age ≥ 65 years and antiplatelet drugs or alcohol use); CrCl and DOACs. Secondly, the incidence of clinical events in patients with different DOACs was compared and adjusted according to the age, gender, CHA2DS2-VASc score, HAS-BLED score, and CrCl of different groups. *P* value < 0.05 is considered statistically significant. All statistical analysis was done using SPSS software version 25.0 (IBM Corporation, Armonk, NY, USA).

## Results

**Fig. 1:**
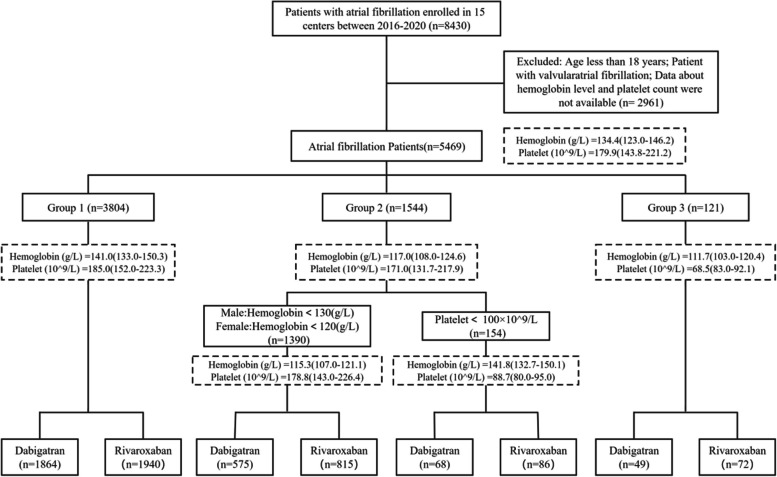
A flowchart of patient enrollment

A total of 5469 patients with NVAF (Mean age 64.6 ± 12.1; 42.1% female) were enrolled in this study, including 3804 patients in Group 1 (Mean age 62.9 ± 11.9; 39.9% female), 1544 patients in Group 2 (Mean age 68.7 ± 11.7; 47.0% female), and 121 patients in Group 3 (Mean age 69.4 ± 10.6; 47.1% female). The proportions of different DOACs dosage groups are shown in Fig. 1. Table 1 shows baseline information for patients. Overall, Groups 2 and 3 had lower CrCl and more complications than Group 1 (all *P* values < 0.001).

**Table 1:** Baseline characteristics of patients with atrial fibrillation stratified by different hemoglobin and platelet levels

	All patients (*n* = 5469)	Group 1 (*n* = 3804)	Group 2 (*n* = 1544)	Group 3 (*n* = 121)	*P* Value
**Baseline characteristics**					
Age (y), mean (SD)	64.6 ± 12.1	62.9 ± 11.9	68.7 ± 11.7	69.4 ± 10.6	< 0.001
Female, n (%)	2300 (42.1)	1517 (39.9)	726 (47.0)	57 (47.1)	< 0.001
Body weight(kg), mean (SD)	66.1 ± 12.2	67.7 ± 12.2	62.7 ± 11.5	58.9 ± 11.6	< 0.001
CHA2DS2-VASc score, mean (SD)	2.2 ± 1.6	2.0 ± 1.5	2.7 ± 1.6	2.3 ± 1.4	< 0.001
HAS-BLED score, mean (SD)	1.4 ± 1.0	1.3 ± 1.0	1.6 ± 1.0	1.5 ± 1.0	< 0.001
**Past medical history, n (%)**					
COPD	109 (2.0)	53 (1.4)	52 (3.4)	4 (3.3)	< 0.001
Coronary heart disease	476 (8.7)	262 (6.9)	199 (12.9)	15 (12.4)	< 0.001
Congestive heart failure	278 (5.1)	141 (3.7)	122 (7.9)	15 (12.4)	< 0.001
Chronic kidney disease	338 (6.2)	195 (5.1)	132 (8.5)	11 (9.1)	< 0.001
Hypertension	2988 (54.6)	2016 (53.0)	921 (59.7)	51 (42.1)	< 0.001
Diabetes mellitus	1032 (18.9)	687 (18.1)	326 (21.1)	19 (15.7)	0.023
Previous stroke	205 (3.7)	104 (2.7)	97 (6.3)	4 (3.3)	< 0.001
Gout	94 (1.7)	47 (1.2)	44 (2.8)	3 (2.5)	< 0.001
Malignancy	77 (1.4)	35 (0.9)	37 (2.4)	5 (4.1)	< 0.001
**Baseline laboratory data, mean(SD)**					
ALT, IU/L	27.3 ± 40.0	27.7 ± 38.6	26.2 ± 43.7	27.6 ± 32.9	0.488
CrCl, mL/min	74.3 ± 30.7	78.5 ± 30.4	65.0 ± 29.1	59.4 ± 28.5	< 0.001
**Baseline medications, n (%)**					
Dabigatran	2548 (46.6)	1861 (48.9)	638 (41.3)	49 (40.5)	< 0.001
110 mg/bid	2469 (45.1)	1796 (47.2)	624 (40.4)	49 (40.5)	< 0.001
150 mg/bid	79 (1.4)	65 (1.7)	14 (0.9)	0 (0)	0.034
Rivaroxaban	2921 (53.4)	1943 (51.1)	906 (58.7)	72 (59.5)	< 0.001
10 mg/qd	790 (14.4)	427 (11.2)	334 (21.6)	29 (24.0)	< 0.001
15 mg/qd	1986 (36.3)	1407 (37.0)	540 (35.0)	39 (32.2)	0.245
20 mg/qd	145 (2.7)	109 (2.9)	32 (2.1)	4 (3.3)	0.237
ACEI/ARB	2380 (43.5)	1659 (43.6)	677 (43.8)	44 (36.4)	0.272
Amiodarone	962 (17.6)	714 (18.8)	229 (14.8)	19 (15.7)	0.002
Beta-blocker	3484 (63.7)	2449 (64.4)	958 (62.0)	77 (63.6)	0.275
CCB	1641 (30.0)	1152 (30.3)	456 (29.5)	33 (27.3)	0.693
Diltiazem	768 (14.0)	529 (13.9)	224 (14.5)	15 (12.4)	0.738
Digoxin	570 (10.4)	373 (9.8)	187 (12.1)	10 (8.3)	0.032
Statins	2729 (49.9)	1872 (49.2)	789 (51.1)	68 (56.2)	0.171

### Association among hemoglobin levels, platelet counts, and clinical events

The median (IQR) of hemoglobin level and platelet count in the whole study population were 134.4(123.0–146.2)g/L and 179.9(143.8–221.2) × 10^9^/L, respectively. Higher hemoglobin levels (per 1 unit increase) were significantly associated with a reduced risk of total bleeding (adjusted odds ratio [aOR] 0.991, 95%CI 0.985–0.998, *P* = 0.010) and major bleeding (aOR 0.978, 95%CI 0.968—0.989, *P* < 0.001), but not thrombosis (aOR 0.988, 95%CI 0.969—1.008, *P* = 0.228). Unlike hemoglobin, platelet levels were not associated with total bleeding (aOR 1.001, 95%CI 0.999–1.003, *P* = 0.363), major bleeding (aOR 1.002, 95%CI 0.999–1.005, *P* = 0.203), or thrombosis (aOR 0.998, 95%CI 0.993–1.004, *P* = 0.564).

### Risks of clinical events in different groups

**Fig. 2:**
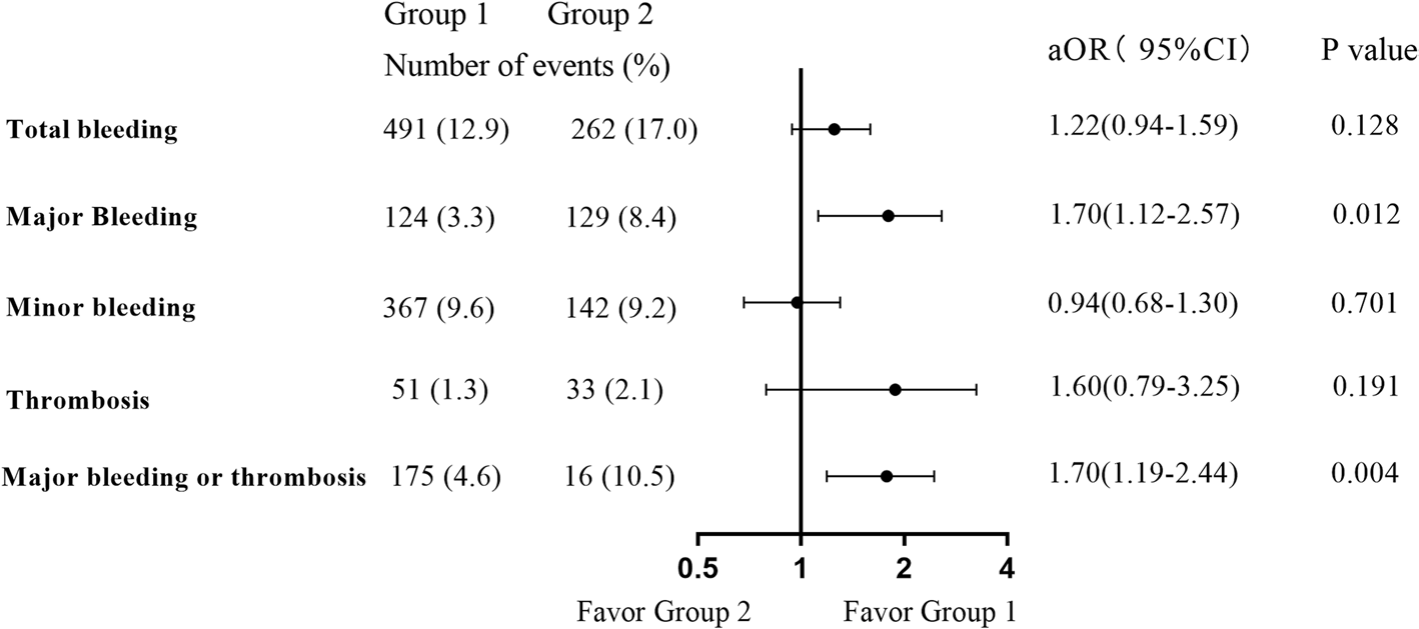
Risks of clinical events of Group 1 versus Group 2

**Fig. 3:**
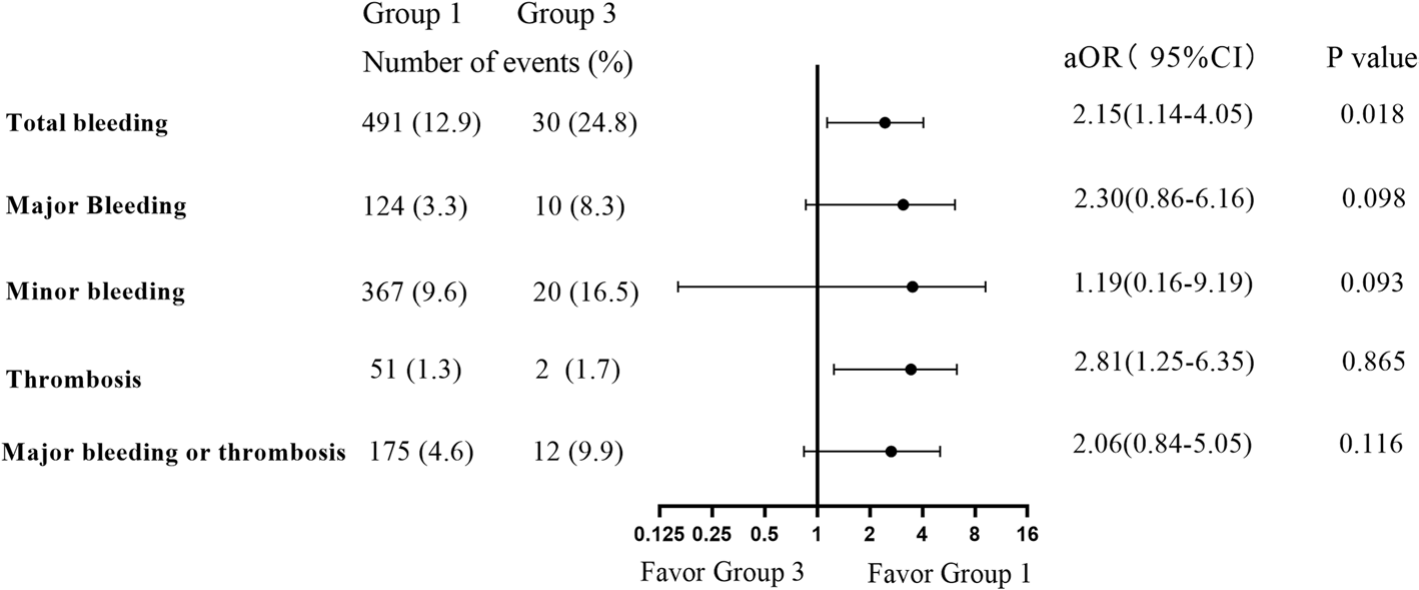
Risks of clinical events of Group 1 versus Group 3

Figures 2 and 3 show the risks of clinical events in different groups. Compared with Group 1, Group 2 had a higher risk of major bleeding (aOR 1.70, 95%CI 1.12–2.57, *P* = 0.012) and composite endpoint of major bleeding and thrombosis (aOR 1.70, 95%CI 1.19–2.44, *P* = 0.004) (Fig. 2). Compared with Group 1, the total bleeding risk of Group 3 (aOR 2.15, 95%CI 1.14–4.05, *P* = 0.018) was higher (Fig. 3).

### Dabigatran versus rivaroxaban

**Fig. 4:**
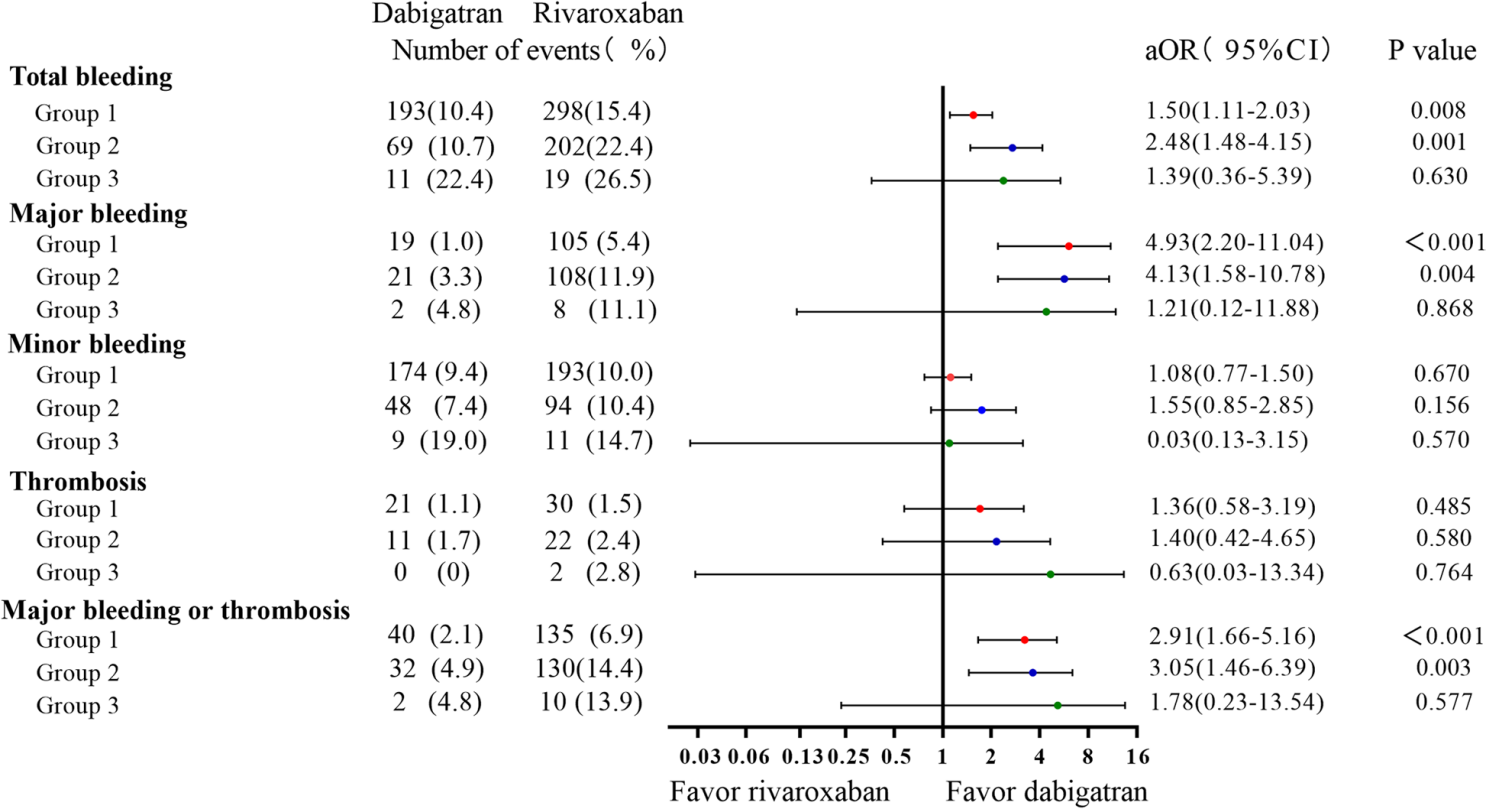
Risks of clinical events of rivaroxaban versus dabigatran treatment in 3 groups

Compared with dabigatran, rivaroxaban was associated with a higher risk of total bleeding in Group 1(aOR 1.50, 95%CI 1.11–2.03, *P* = 0.008) and Group 2(aOR 2.48, 95%CI 1.48–4.15, *P* = 0.001), but not in Group 3 (aOR 1.39, 95%CI 0.36–5.39, *P* = 0.630). Compared with dabigatran, rivaroxaban was associated with a higher risk of major bleeding in Group 1(aOR 4.93, 95%CI 2.20–11.04, *P* < 0.001) and Group 2(aOR 4.13, 95%CI 1.58–10.78, *P* = 0.004), but not in Group 3 (aOR 1.21, 95%CI 0.12–11.88, *P* = 0.868). Compared with dabigatran, rivaroxaban was associated with a higher composite endpoint risk of major bleeding and thrombosis in Group 1 (aOR 2.91, 95%CI 1.66–5.16, *P* < 0.001) and Group 2 (aOR 3.05, 95%CI 1.46–6.39, *P* = 0.003), but not in Group 3 (aOR 1.78, 95%CI 0.23–13.54, *P* = 0.577) (Fig. 4).

**Fig. 5:**
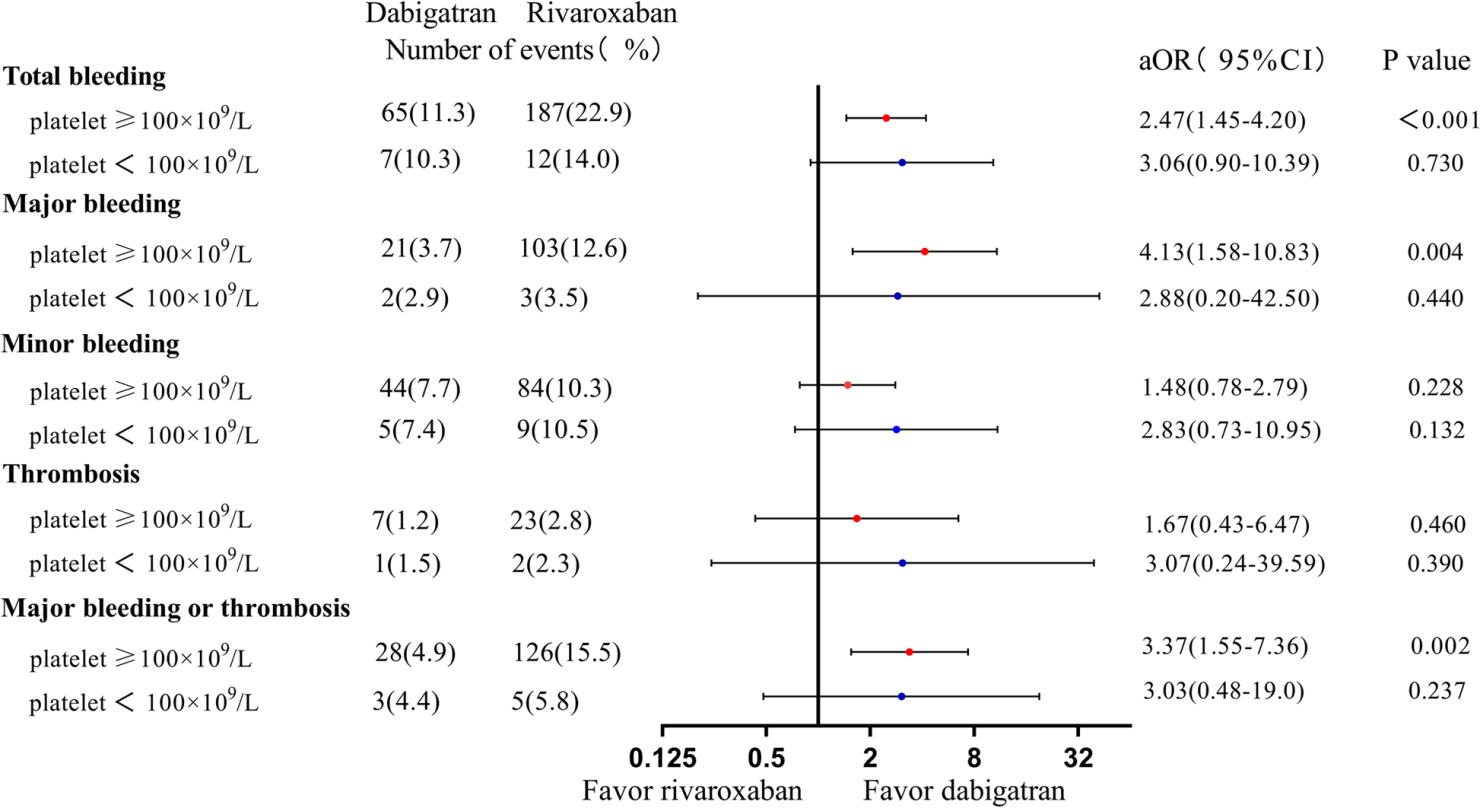
Risks of clinical events of rivaroxaban versus dabigatran in subgroups of group 2

We compared dabigatran and rivaroxaban for subgroup patients with either anemia (*n* = 1390) or thrombocytopenia (*n* = 154) among Group 2. As Fig. 5 shows, the use of rivaroxaban was consistently associated with a higher risk of total bleeding (aOR 2.47, 95%CI 1.45–4.20, *P* < 0.001), major bleeding (aOR 4.13, 95%CI 1.58–10.83, *P* = 0.004), and composite risk of major bleeding or thrombosis in subgroups with anemia (aOR 3.37, 95%CI 1.55–7.36, *P* = 0.002).

## Discussion

In this study, we investigated the association of clinical event risk with hemoglobin levels and platelet counts in patients with AF and the clinical outcomes of different DOACs in different hemoglobin and platelet stratification. Our main findings are as follows: 1) Higher hemoglobin level is related to the reduced risk of total bleeding and major bleeding, while platelet count is not related to any events; 2) Patients with anemia (hemoglobin male < 130 g/L; female < 120 g/L) or thrombocytopenia (platelet count < 100 × 10^9^/L) are at high risk, and the risk of major bleeding, composite risk of major bleeding or thrombosis is higher; 3) Compared with dabigatran, rivaroxaban was associated with a higher risk of total bleeding, major bleeding, and major bleeding or thrombosis in AF patients with anemia or thrombocytopenia, regardless of other factors that may affect the clinical outcome, such as dosage and complications; 4) Compared with rivaroxaban, dabigatran has no significant correlation with better clinical results in patients with anemia and thrombocytopenia.

### Relationship between hemoglobin level or platelet count and clinical outcomes

Previous reports have shown that anemia is associated with an increased risk of bleeding [15, 16, 31], so anemia is included in most bleeding risk scores [8]. In the current study, we also observed that the increase in hemoglobin level would reduce the risk of total bleeding and major bleeding. On the contrary, much less evidence exists about the association between anemia and stroke/systemic embolism. Kodani et al. [18] studied 6536 Japanese patients with AF and found that hemoglobin levels were not associated with thrombosis. Similar to the study by Kodani et al., our study also showed that hemoglobin levels were not associated with thrombosis. However, another analysis of a large cohort of AF patients reported that higher hemoglobin levels were associated with a higher risk of ischemic stroke/systemic embolism [16]. Although an association between anemia and stroke risk cannot be excluded, our analysis suggests that anemia is more strongly associated with bleeding than stroke.

Evidence shows that the association between anemia and low platelet function is related to decreased platelet marginalization tendency [32]. Platelets play an essential role in primary aggregation. Therefore, platelet activity is related to the incidence of thrombosis and hemorrhagic events. It is reported that the platelet size measured by the average platelet volume is related to the incidence of stroke [33, 34] or coronary artery disease [35], and the platelet count showing bone marrow platelet production is also related to the occurrence of stroke or bleeding events [he Effect of Hematocrit on Platelet Adhesion: Experiments and Simulations]. There are few data about the role of platelet count as a prognostic predictor of AF patients, and there are conflicting results. The research results of Yeh et al. [16] and Park et al. [19] show that a lower platelet count is associated with a lower risk of stroke and a higher risk of bleeding events. However, Kodani et al. [18] reported that platelet levels did not affect the outcome of thrombosis or bleeding in AF patients. Similar to the study by Kodani et al., our study also showed that platelet levels were not associated with thrombosis or bleeding. Higher rates of OAC use and lower rates of cumulative stroke/bleeding events in our study and Kodani et al. compared to those in Park et al. and Yeh et al. may account for the present results.

### DOACs in patients with anemia or thrombocytopenia

The underuse of OACs for stroke prevention is a common problem in the Asia–Pacific region [36]. Compared with Western populations, Asian AF patients have a smaller body size and a higher risk of bleeding, such as intracranial hemorrhage [37]. Concerns about bleeding may further prevent using OACs in patients with anemia or thrombocytopenia. Therefore, the selection of appropriate OACs is crucial for AF patients. Because a significant proportion of AF patients with anemia or thrombocytopenia do not qualify for the four major clinical trials of DOACs [23–26], the choice of anticoagulants in this population has been confusing. Wang and colleagues separately compared the safety and efficacy of DOACs with warfarin in AF patients with anemia or thrombocytopenia [27, 28]. They showed that DOACs were associated with a lower risk of bleeding than warfarin in AF patients with hemoglobin < 10 g/dL or platelet counts < 100 × 10^3^/µL. However, there was no difference in risk for ischemic stroke/systemic embolism or death. Our study further found that dabigatran was associated with a lower total bleeding risk and composite risk of major bleeding or thrombosis compared with rivaroxaban, and dabigatran is a reasonable choice for stroke prevention in AF patients with anemia or thrombocytopenia. These findings fill the knowledge gap about the safety and effectiveness of DOACs in stroke prevention in AF patients with anemia or thrombocytopenia.

Good compliance with OACs is essential to realize the prognostic benefits of anticoagulation. Considering the short half-life of DOACs, the clinical consequences of non-adherence may be more profound [38]. However, long-term non-adherence to DOAC treatment is very serious in China. The results of a cohort study show that only 35% of the patients in Guangzhou, where the economic level of China is relatively high, continue to receive DOAC treatment for more than one year [39]. Because of the increased risk of bleeding in patients with AF complicated with anemia or thrombocytopenia, anticoagulation therapy is more likely to be interrupted, and they need more extensive follow-up when receiving anticoagulation therapy. In this cohort, 33.4% of patients were complicated with anemia and/or thrombocytopenia before starting oral anticoagulant therapy. Before starting DOAC treatment, these patients should carefully assess their bleeding risk, investigate their bleeding history and clinical conditions that may lead to bleeding (such as peptic ulcer disease, impaired renal function, or liver function), and correct them reversibly. Drugs that may increase the risk of major bleeding (such as non-steroidal anti-inflammatory drugs or antiplatelet drugs) should be avoided or used after weighing the advantages and disadvantages.

### Strengths and limits

Our study’s strengths include using a large, real-world population sample of 15 hospitals from different provinces in China, covering most of China, making our findings somewhat representative. We also obtained extensive laboratory data, including data on baseline hemoglobin, platelet count, renal function, and liver function that were available before oral anticoagulation initiation and generally unavailable in administrative registries. To our knowledge, this is the only study comparing different DOACs for stroke prevention in AF patients with anemia or thrombocytopenia.

The current study also has some limitations. First, in our multicenter cohort, 2293 AF patients without hemoglobin or platelet data were excluded from the analysis. Whether the study sample can be representative of the population as a whole remains uncertain; therefore, some bias may be present. Second, the three groups’ baseline characteristics differed due to the study’s retrospective nature. Although statistical adjustments were made for several important parameters that could influence clinical outcomes, we cannot rule out the possibility of bias by unmeasured confounders. Finally, the number of patients in group 3 was small. The analysis in this group may not have been sufficiently robust, limiting the generalizations of the results, and further studies in this population are warranted.

## Conclusion

Higher hemoglobin levels are associated with a reduced risk of total bleeding and major bleeding in patients with AF. Compared with AF patients, AF patients with anemia or thrombocytopenia have a higher risk of major bleeding, a composite endpoint of major bleeding and thrombosis. Dabigatran was associated with better clinical outcomes than rivaroxaban in patients with anemia or thrombocytopenia but not in those with anemia and thrombocytopenia.

### Supplementary Information

**Additional file 1: Supplemental Table 1. **List of 15 multi-center hospitals.** Supplemental Figure 1. **Sub-center Distribution Map.

## Data Availability

The data that support the findings of this study are available from the corresponding author upon reasonable request.
