# Supplementary material for: Safety and efficacy of direct oral anticoagulants in stroke prevention in patients with atrial fibrillation complicated with anemia and/or thrombocytopenia: a retrospective cohort study
Source: Thromb J. 2023 Nov 21;21:118. doi: 10.1186/s12959-023-00563-7 (PMC10662166; doi:10.1186/s12959-023-00563-7)
Supplement: Supplementary file 1 — Additional file 1: Supplemental Table 1. List of 15 multi-center hospitals. Supplemental Figure 1. Sub-center Distribution Map. [file 12959_2023_563_MOESM1_ESM.docx]

**Supplementary materials**

**Supplemental Table 1.** List of 15 multi-center hospitals

**Supplemental Figure 1.** Sub-center Distribution Map

**Supplemental Table 1** List of 15 multi-center hospitals

| Number | Institutes | Investigators |
| --- | --- | --- |
| 1 | Shanxi Bethune Hospital | Ruijuan Li |
| 2 | Suining Central Hospital, Suining | Ping Gu |
| 3 | First Affiliated Hospital of Xi'an Jiaotong University | Qiaowei Zheng |
| 4 | People’s Hospital of He’nan University of Chinese Medicine | Xiumei Liu |
| 5 | Affiliated Fuzhou First Hospital of Fujian Medical University | Hengfen Dai |
| 6 | Pingtan County General Laboratory Area Hospital | Xiangsheng Lin |
| 7 | Huaihe Hospital of Henan University, kaifeng | Yuxin Liu |
| 8 | Shengjing hospital of China Medical University | Xiaoming Du |
| 9 | Department of Pharmacy, the First Affiliated Hospital of Bengbu Medical College | Jun Su |
| 10 | The first people‘s Hospital of Changde City | Wang Zhang |
| 11 | Affiliated Qingdao Third People's Hospital | Min Zhang |
| 12 | The Second Affiliated Hospital of Soochow University | Zhu Zhu |
| 13 | Zhangzhou affiliated Hospital of Fujian Medical University | Xiaohong Huang |
| 14 | Wuhan Asian Heart Hospital | Nianxu Huang |
| 15 | Fujian Medical University Union Hospital | Jinhua Zhang |


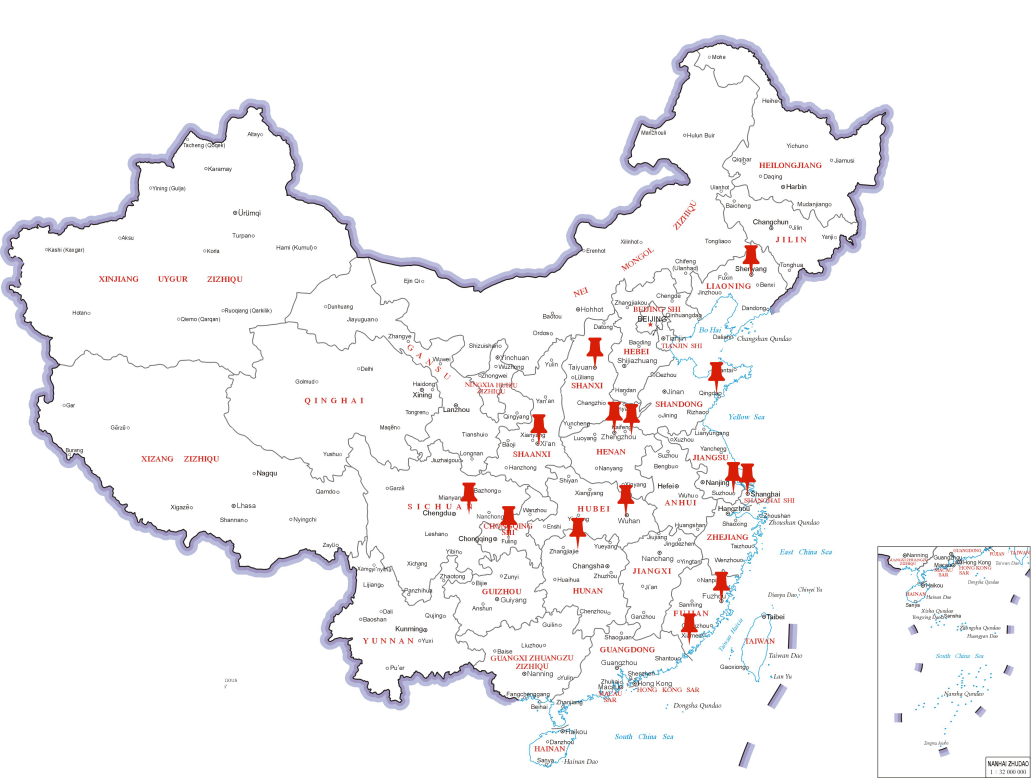


**Supplemental Figure 1.** Sub-center Distribution Map
